# Phenotypic Age Acceleration as a Mediator in Thyroid Hormone–Related Cardiovascular Risk Among the Elderly

**DOI:** 10.1155/crp/1206757

**Published:** 2026-06-28

**Authors:** Minmin Wen, Yanjun Hou, Kaijia Shi, Jiaxin Zuo, Cheng Zhang, Shuya Zhang, Zhihua Shen, Wei Jie

**Affiliations:** ^1^ Key Laboratory of Tropical Translational Medicine of Ministry of Education & Hainan Provincial Key Laboratory for Tropical Cardiovascular Diseases Research, School of Public Health, Hainan Medical University, Haikou, 571199, China, hainmc.edu.cn; ^2^ Department of Cardiovascular Surgery, The Second Affiliated Hospital, Hainan Medical University, Haikou, 570100, China, hainmc.edu.cn; ^3^ Department of Pathophysiology, School of Basic Medicine Sciences, Guangdong Medical University, Zhanjiang, 524023, China, gdmu.edu.cn

**Keywords:** cardiovascular disease, free triiodothyronine, NHANES, phenotypic age acceleration, thyroid hormone, total triiodothyronine

## Abstract

**Background:**

The association between thyroid hormones and cardiovascular disease (CVD) has been widely examined; however, the role of biological aging in this relationship remains unclear. This study systematically investigates the mediating role of phenotypic age acceleration (PhenoAgeAccel) in the relationship between thyroid hormones and CVD.

**Methods:**

The data were sourced from the 2007–2010 cycles of the National Health and Nutrition Examination Survey (NHANES). Analyses included logistic regression, linear regression, restricted cubic splines, subgroup analyses, and mediation analysis to assess the role of PhenoAgeAccel.

**Results:**

Among participants aged ≥ 60 years, elevated free triiodothyronine (FT3) concentrations were linked to a reduced risk of CVD (adjusted odds ratio [aOR] = 0.483; 95% CI: 0.273–0.856; *p* = 0.013). Similarly, total triiodothyronine (TT3) levels were associated with a decreased CVD risk in those aged 60 and above (aOR = 0.988; 95% CI: 0.980–0.995; *p* = 0.013). Mediation analysis showed that PhenoAgeAccel mediated 21.13% of the FT3–CVD association and 23.41% of the TT3–CVD association in older adults.

**Conclusion:**

This study identifies an age‐dependent relationship between FT3, TT3, and CVD and reveals that PhenoAgeAccel partially mediates this association. These findings suggest that interventions targeting thyroid function and biological aging may offer novel strategies for CVD prevention and management in older populations.

## 1. Introduction

The thyroid is a crucial endocrine gland that regulates numerous bodily processes, including metabolism, growth, tissue development, and reproduction [1]. The principal hormones associated with how the thyroid functions include triiodothyronine (T3), thyroxine (T4), and thyroid‐stimulating hormone (TSH) [2]. Studies have shown that hypothyroidism significantly increases the risk of cardiovascular disease (CVD)–related mortality [3]. Additionally, both subclinical hypothyroidism and subclinical hyperthyroidism are associated with higher risks of cardiovascular morbidity and all‐cause mortality [4]. Notably, higher free thyroxine (FT4) and lower free triiodothyronine (FT3) levels are correlated with increased risks of all‐cause and CVD mortality [5]. Thus, thyroid hormone (TH) levels are significantly linked to cardiovascular and cerebrovascular mortality [6, 7].

Although aging is an irreversible biological process, it is a major risk factor for chronic diseases and death [8]. The thyroid gland plays an important regulatory role in human aging [9]. Emerging evidence suggests that chronological age alone is insufficient to capture individual variability in biological aging trajectories and overall health status [10]. Accordingly, Levine and colleagues proposed a new biological age metric called phenotypic age (PhenoAge), based on large cohorts such as National Health and Nutrition Examination Survey (NHANES) [11]. Its derivative, PhenoAgeAccel, measures accelerated biological aging using clinical biochemical parameters. It reflects aging characteristics at multiple cellular levels, helping to identify poor health outcomes from multiorgan diseases [12]. Compared with chronological age, biological age offers an opportunity for early intervention and disease prevention. It has significant clinical utility in managing age‐related diseases in the elderly [13]. Therefore, PhenoAge and PhenoAgeAccel are increasingly applied in epidemiological studies of aging‐related CVD [14, 15].

Recent studies have shown an age‐dependent link between thyroid function and biological aging. A study by Liu et al., using NHANES data, revealed that the association between FT3 and PhenoAgeAccel varies with age: Higher FT3 levels were associated with increased PhenoAgeAccel risk in adults aged < 60 years, but with decreased risk in those aged ≥ 60 years [16]. Although associations between THs and CVD and between THs and PhenoAgeAccel have been suggested, whether PhenoAgeAccel mediates the TH–CVD relationship remains unexplored.

This study, therefore, aims to systematically investigate the association between THs and CVD events and to examine the mediating role and effect size of PhenoAgeAccel.

## 2. Methods

### 2.1. Data Sources and Study Population

**FIGURE 1:**
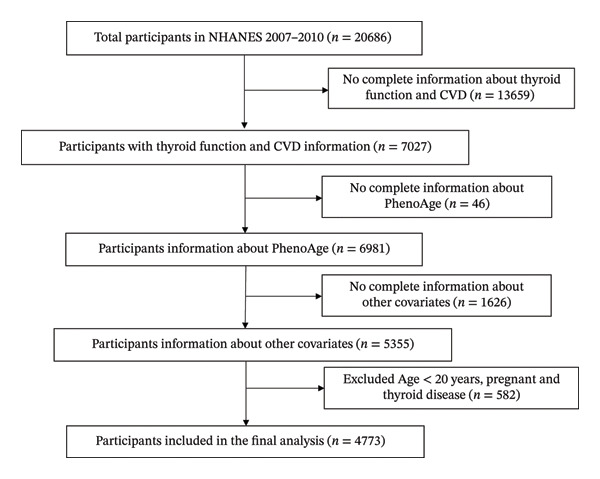
Flowchart of participant screening from NHANES 2007–2010.

All NHANES data are publicly available at https://www.cdc.gov/nchs/nhanes/. This study used NHANES continuous data from 2007 to 2010, including 20,686 participants. After screening for complete data on thyroid function, PhenoAge metrics, and self‐reported CVD, 6981 participants were eligible. Subsequently, 1626 participants with missing covariate data were excluded, and 582 participants with a history of thyroid disease, pregnant women, or individuals aged < 20 years were removed. As shown in Figure 1, the final analytic sample comprised 4773 participants.

### 2.2. Study Variables

PhenoAge was calculated based on 10 key biomarkers associated with aging: chronological age, creatinine, albumin, glucose, lymphocyte percentage, C‐reactive protein, mean corpuscular volume, alkaline phosphatase (ALP), red blood cell distribution width (RDW), and white blood cell count [17]. Chronological age was derived from demographic variables, while the other nine biomarkers were sourced from laboratory data within the NHANES database. PhenoAgeAccel was defined as the residual obtained from regressing PhenoAge on chronological age, representing the extent of accelerated biological aging in years compared to chronological age [18]. The formula for PhenoAge is given as follows:

phenotypic age = 141.50 + Ln[−0.00553 × In(1 − M)]/0.09165, where M  = 1 − exp  (−1.51714 × exp(*x* *b*)/0.0076927), and xb = −19.907−0.0336 × albumin +0.0095 × creatinine +0.1953 × glucose +0.0954 × ln(CRP) −0.0120 × lymphocyte percent +0.0268 × mean cell volume +0.3306 × RDW +0.00188 × ALP +0.0554 × white blood cell count +0.0804 × chronological age [12].

### 2.3. Covariates

Covariates included age, sex, race/ethnicity, education, marital status, body mass index (BMI), hypertension, diabetes, alcohol consumption, smoking status, vigorous work activity, vigorous recreational activities, average daily caloric intake, and urine iodine concentration (UIC). Race/ethnicity was grouped into five categories following NHANES criteria: Mexican American, Other Hispanic, non‐Hispanic White, non‐Hispanic Black, and Other. Education was dichotomized as high school or less vs. beyond high school. Marital status included six types: married, never married, living with a partner, divorced, widowed, and separated. Hypertension, diabetes, vigorous work activity, and vigorous recreational activities were binary variables. Average daily caloric intake was derived from two 24‐h dietary recalls [19]. Alcohol consumption was defined as having consumed ≥ 12 drinks in the past year. Smoking was defined as having smoked ≥ 100 cigarettes in a lifetime. UIC was categorized as < 100, 100–300, and ≥ 300 μg/L. CVD was identified based on self‐reported diagnoses of stroke, angina, myocardial infarction, congestive heart failure, or coronary heart disease [20].

### 2.4. Statistical Analysis

We used analytical methods appropriate for the NHANES complex survey design, accounting for stratification and weighting. Categorical variables were summarized as percentages, and continuous variables as means ± standard deviations. Group comparisons were made using chi‐square tests for categorical variables and one‐way ANOVA for continuous variables. All tests were two‐sided, with *p* < 0.05 considered statistically significant. To assess whether PhenoAgeAccel mediates the relationship between FT3/TT3 and CVD, we considered three pathways: (A) FT3/TT3 ⟶ PhenoAgeAccel; (B) PhenoAgeAccel ⟶ CVD; (C) FT3/TT3 ⟶ CVD. For Pathway A, multiple linear regression was used, with results reported as beta coefficients (*β*) and 95% confidence intervals (CI) per one‐SD increase in hormone levels. For Pathways B and C, logistic regression was used, with results as odds ratios (OR) and 95% CI. Mediation analysis was conducted to estimate the proportion of the total effect mediated by PhenoAgeAccel [21]. All analyses were performed in R Version 4.5.0.

## 3. Results

### 3.1. Study Participants and Baseline Characteristics

Table 1 summarizes the characteristics of NHANES participants stratified by CVD status. The analysis included 4773 U.S. adults. Among them, 505 (10.58%) had CVD, and 4268 (89.42%) did not. Significant differences (*p* < 0.05) were observed for age, sex, race/ethnicity, education, marital status, BMI, hypertension, diabetes, smoking, vigorous work activity, vigorous recreational activities, caloric intake, UIC, PhenoAge, PhenoAgeAccel, TSH, FT3, FT4, and TT3. Compared with non‐CVD participants, those with CVD were older and more likely to be male and had lower educational attainment, higher BMI, and higher prevalence of hypertension, diabetes, and smoking, as well as higher PhenoAge and PhenoAgeAccel.

**TABLE 1:** Basic characteristics of the participants in this study.

	Total			20–59 years			≥ 60 years		
	Non‐CVD (*n* = 4268)	CVD (*n* = 505)	*p*	Non‐CVD (*n* = 3010)	CVD (*n* = 127)	*p*	Non‐CVD (*n* = 1258)	CVD (*n* = 378)	*p*
Sex, *n* (%)			< 0.001			0.581			< 0.001
Male	2183 (51.15)	326 (64.55)		1538 (51.10)	75 (59.06)		645 (51.27)	251 (66.40)	
Female	2085 (48.85)	179 (35.45)		1472 (48.90)	52 (40.94)		613 (48.73)	127 (33.60)	
Age, years	45.24 ± 15.63	64.02 ± 13.03	< 0.001	39.46 ± 11.23	48.87 ± 8.10	< 0.001	68.52 ± 6.61	71.89 ± 6.55	< 0.001
Race, *n* (%)			0.005			0.168			0.675
Mexican American	772 (18.09)	51 (10.10)		611 (20.30)	18 (14.17)		161 (12.80)	33 (8.73)	
Other Hispanic	484 (11.34)	35 (6.93)		355 (11.79)	12 (9.45)		129 (10.25)	23 (6.08)	
Non‐Hispanic White	2037 (47.73)	302 (59.80)		1343 (44.62)	63 (49.61)		694 (55.17)	239 (63.23)	
Non‐Hispanic Black	808 (18.93)	106 (20.99)		565 (18.77)	30 (23.62)		243 (19.32)	76 (20.11)	
Other races	167 (3.91)	11 (2.18)		136 (4.52)	4 (3.15)		31 (2.46)	7 (1.85)	
Education, *n* (%)			< 0.001			0.019			0.019
≤ High school diploma	2229 (52.23)	304 (60.20)		1525 (50.66)	77 (60.63)		704 (55.96)	227 (60.05)	
> High school diploma	2039 (47.77)	201 (39.80)		1485 (49.34)	50 (39.37)		554 (44.04)	151 (39.95)	
Marriage, *n* (%)			< 0.001			0.003			0.219
Married	2316 (54.27)	289 (57.23)		1568 (52.10)	67 (52.76)		748 (59.46)	222 (58.73)	
Widowed	284 (6.65)	91 (18.02)		44 (1.46)	5 (3.94)		240 (19.08)	86 (22.75)	
Divorced	461 (10.80)	61 (12.08)		293 (9.73)	18 (14.17)		168 (13.35)	43 (11.38)	
Separated	132 (3.09)	15 (2.97)		112 (3.72)	8 (6.30)		20 (1.59)	7 (1.85)	
Never married	731 (17.13)	30 (5.94)		680 (22.59)	15 (11.81)		51 (4.05)	15 (3.97)	
Living with a partner	344 (8.06)	19 (3.76)		313 (10.40)	14 (11.02)		31 (2.46)	5 (1.32)	
UIC, *n* (%)			0.015			0.931			0.031
< 100 μg/L	1349 (31.61)	135 (26.73)		994 (33.02)	43 (33.86)		355 (28.22)	92 (24.34)	
100–300 μg/L	2114 (49.53)	232 (45.94)		1478 (49.10)	59 (46.46)		636 (50.56)	173 (45.77)	
≥ 300 μg/L	805 (18.86)	138 (27.33)		538 (17.87)	25 (19.69)		267 (21.22)	113 (29.89)	
Energy intake (kcal/day)	2158.10 ± 831.02	1812.24 ± 741.86	< 0.001	2242.83 ± 854.01	1901.56 ± 920.84	< 0.001	1816.79 ± 624.18	1765.87 ± 626.42	0.204
BMI (kg/m^2^)	28.42 ± 6.29	29.92 ± 6.64	< 0.001	28.35 ± 6.42	31.41 ± 8.29	0.002	28.74 ± 5.72	29.15 ± 5.46	0.261
TSH (mIU/L)	1.94 ± 1.77	2.05 ± 1.37	0.043	1.86 ± 1.58	1.94 ± 1.26	0.629	2.29 ± 2.35	2.11 ± 1.42	0.154
FT3 (pg/mL)	3.21 ± 0.47	3.06 ± 0.37	< 0.001	3.25 ± 0.49	3.27 ± 0.32	0.423	3.03 ± 0.32	2.95 ± 0.35	0.002
FT4 (pmol/L)	9.96 ± 1.69	10.39 ± 2.23	0.022	9.90 ± 1.66	10.10 ± 1.99	0.685	10.21 ± 1.77	10.54 ± 2.33	0.222
TT3 (ng/dL)	114.43 ± 22.38	106.12 ± 23.60	< 0.001	116.28 ± 22.52	116.23 ± 23.34	0.958	106.94 ± 20.16	100.86 ± 22.01	< 0.001
TT4 (μg/dL)	7.72 ± 1.50	7.81 ± 1.62	0.510	7.70 ± 1.49	7.76 ± 1.39	0.757	7.79 ± 1.53	7.84 ± 1.72	0.903
Hypertension, *n* (%)			< 0.001			< 0.001			< 0.001
Yes	1271 (29.78)	365 (72.28)		591 (19.63)	92 (72.44)		680 (54.05)	273 (72.22)	
No	2997 (70.22)	140 (27.72)		2419 (80.37)	35 (27.56)		578 (45.95)	105 (27.78)	
Diabetes, *n* (%)			< 0.001			< 0.001			< 0.001
Yes	395 (9.25)	158 (31.29)		173 (5.75)	37 (29.13)		222 (17.65)	121 (32.01)	
No	3873 (90.75)	347 (68.71)		2837 (94.25)	90 (70.87)		1036 (82.35)	257 (67.99)	
Drink, *n* (%)			0.145			0.401			0.961
Yes	3097 (72.56)	356 (70.50)		2264 (75.22)	102 (80.31)		833 (66.22)	254 (67.20)	
No	1171 (27.44)	149 (29.50)		74 (24.78)	25 (19.69)		425 (33.78)	124 (32.80)	
Smoke, *n* (%)			< 0.001			< 0.001			0.060
Yes	1969 (46.13)	320 (63.37)		1313 (43.62)	84 (66.14)		656 (52.15)	236 (62.43)	
No	2299 (53.87)	185 (36.63)		1697 (56.38)	43 (33.86)		602 (47.85)	142 (37.57)	
Vigorous work activity, *n* (%)			< 0.001			0.379			0.017
Yes	927 (21.72)	53 (10.50)		749 (24.88)	22 (17.32)		178 (14.15)	31 (8.20)	
No	3341 (78.28)	452 (89.50)		2261 (75.12)	105 (82.68)		1080 (85.85)	347 (91.80)	
Vigorous recreational activities, *n* (%)			< 0.001			0.012			0.034
Yes	922 (21.60)	33 (6.53)		812 (26.98)	17 (13.39)		110 (8.74)	16 (4.23)	
No	3346 (78.40)	472 (93.47)		2198 (73.02)	110 (86.61)		1148 (91.26)	362 (95.77)	
PhenoAge (years)	40.05 ± 17.49	63.66 ± 16.96	< 0.001	34.11 ± 13.22	46.95 ± 12.63	< 0.001	63.96 ± 11.00	72.34 ± 11.57	< 0.001
PhenoAgeAccel, *n* (%)			< 0.001			0.094			< 0.001
Yes	1935 (45.34)	318 (62.97)		1408 (46.78)	77 (60.63)		527 (41.89)	241 (63.76)	
No	2333 (54.66)	187 (37.03)		1602 (53.22)	50 (39.37)		731 (58.11)	137 (36.24)	

*Note:* Continuous variables were presented as mean ± SD. Categorical variables were presented as *n* (%). CVD, cardiovascular disease; FT3, free triiodothyronine; FT4, free thyroxine; TT3, total triiodothyronine; TT4, total thyroxine.

Abbreviations: BMI, body mass index; PhenoAge, phenotypic age; PhenoAgeAccel, phenotypic age acceleration; TSH, thyroid‐stimulating hormone; UIC, urine iodine concentration.

In the 20‐ to 59‐year age group, the CVD group was older and had a higher proportion of males, lower education, higher prevalence of hypertension, diabetes, and smoking, and higher PhenoAge (all *p* < 0.05). PhenoAgeAccel did not differ significantly between groups (*p* = 0.094). FT3 and TT3 levels showed no significant differences. In the ≥ 60‐year age group, consistent with the younger group, the CVD group was older and had a higher proportion of males, lower education, higher prevalence of hypertension, diabetes, and smoking, and higher PhenoAge (all *p* < 0.05). Notably, PhenoAgeAccel differed significantly between groups (*p* < 0.001). FT3 (*p* = 0.002) and TT3 (*p* < 0.001) levels were significantly lower in the CVD group.

### 3.2. The Relationship Between FT3 and TT3 Levels and CVD

**FIGURE 2:**
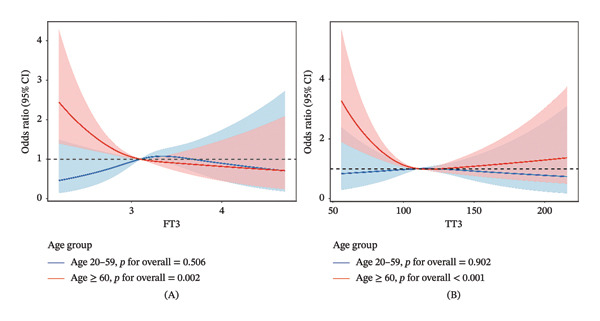
Nonlinear association between thyroid function and CVD. (A) FT3 and CVD; (B) TT3 and CVD. Adjusted for sex, race, education, marital status, BMI, smoking, alcohol, hypertension, vigorous work activity, vigorous recreational activities, average daily caloric intake, UIC, and diabetes.

Weighted multivariate logistic regression showed that among participants aged ≥ 60 years, the analysis revealed a significant inverse association between FT3 levels and the risk of developing CVD (Table 2). In the fully adjusted model, OR = 0.483 (95% CI: 0.273–0.856; *p* = 0.013). This association was consistent across models: Model 1 (OR = 0.473; 95% CI: 0.273–0.820; *p* = 0.007) and Model 2 (OR = 0.400; 95% CI: 0.229–0.731; *p* = 0.005). Similarly, higher TT3 levels were associated with lower CVD risk in the fully adjusted model (OR = 0.988; 95% CI: 0.980–0.995; *p* = 0.013), and this association was significant in Model 1 (OR = 0.985; 95% CI: 0.977–0.993; *p* < 0.001) and Model 2 (OR = 0.985; 95% CI: 0.978–0.992; *p* < 0.001). No significant associations were found in the younger age group or in the overall sample. Restricted cubic spline (RCS) logistic regression revealed a significant overall association between FT3 and CVD among participants aged ≥ 60 years (*p* for overall = 0.002), and similarly for TT3 (*p* for overall < 0.001) (Figure 2).

**TABLE 2:** Associations of FT3 and TT3 with CVD among participants.

		Model^1^		Model^2^		Model^3^	
		OR (95% CI)	*p*	OR (95% CI)	*p*	OR (95% CI)	*p*
FT3	Total	0.312 (0.207, 0.470)	< 0.001	1.010 (0.711, 1.447)	0.934	1.060 (0.751, 1.498)	0.696
	20–59 years	1.051 (0.874, 1.264)	0.597	1.082 (0.894, 1.309)	0.400	1.142 (0.974, 1.339)	0.102
	≥ 60 years	0.473 (0.273, 0.820)	0.007	0.400 (0.229, 0.731)	0.005	0.483 (0.273, 0.856)	0.013

TT3	Total	0.981 (0.972, 0.991)	< 0.001	0.996 (0.988, 1.004)	0.336	0.998 (0.991, 1.006)	0.638
	20–59 years	1.000 (0.986, 1.014)	0.987	0.999 (0.988, 1.009)	0.801	1.000 (0.992, 1.008)	0.972
	≥ 60 years	0.985 (0.977, 0.993)	< 0.001	0.985 (0.978, 0.992)	< 0.001	0.988 (0.980, 0.995)	0.013

*Note:* FT3, free triiodothyronine; TT3, total triiodothyronine.

Abbreviations: CI, confidence interval; OR, odds ratio.

^1^Crude model.

^2^Adjusted for sex, age, race/ethnicity, education level, and marital status.

^3^Adjusted for sex, age, race/ethnicity, education level, marital status, BMI, alcohol drinking status, smoking status, diabetes, hypertension, vigorous work activity, vigorous recreational activities, average daily caloric intake, and UIC.

### 3.3. The Association Between FT3, TT3, and PhenoAgeAccel

Multiple linear regression showed that both FT3 and TT3 were negatively associated with PhenoAgeAccel (Table 3). For FT3, the per‐SD *β* in the crude model was −0.866 (95% CI: −1.287 to −0.445); after full adjustment, *β* = −1.132 (95% CI: −1.539 to −0.725; *p* < 0.001). For TT3, the crude *β* was −0.038 (95% CI: −0.048 to −0.028); after full adjustment, *β* = −0.045 (95% CI: −0.054 to −0.036; *p* < 0.001).

**TABLE 3:** Associations of FT3 and TT3 with PhenoAgeAccel among participants.

	Model^1^		Model^2^		Model^3^	
	*β* (95% CI)	*p*	*β* (95% CI)	*p*	*β* (95% CI)	*p*
FT3	−0.866 (−1.287, −0.445)	< 0.001	−1.099 (−1.541, −0.658)	< 0.001	−1.132 (−1.539, −0.725)	< 0.001
TT3	−0.038 (−0.048, −0.028)	< 0.001	−0.043 (−0.053, −0.033)	< 0.001	−0.045 (−0.054, −0.036)	< 0.001

*Note:* FT3, free triiodothyronine; TT3, total triiodothyronine.

Abbreviation: CI, confidence interval.

^1^Crude model.

^2^Adjusted for sex, age, race/ethnicity, education level, and marital status.

^3^Adjusted for sex, age, race/ethnicity, education level, marital status, BMI, alcohol drinking status, smoking status, diabetes, hypertension, vigorous work activity, vigorous recreational activities, average daily caloric intake, and UIC.

### 3.4. Relationship Between PhenoAgeAccel and CVD

PhenoAgeAccel showed a consistent positive association with CVD risk across all models (Table 4). In the crude model, OR = 1.060 (95% CI: 1.045–1.066; *p* < 0.001). After full adjustment, OR = 1.020 (95% CI: 1.013–1.038; *p* = 0.002).

**TABLE 4:** Associations between PhenoAgeAccel and CVD among participants.

Outcome	Model^1^		Model^2^		Model^3^	
	OR (95% CI)	*p*	OR (95% CI)	*p*	OR (95% CI)	*p*
CVD	1.060 (1.045, 1.066)	< 0.001	1.050 (1.032, 1.060)	< 0.001	1.020 (1.013, 1.038)	0.002

*Note:* CVD, cardiovascular disease.

Abbreviations: CI, confidence interval; OR, odds ratio.

^1^Crude model.

^2^Adjusted for sex, age, race/ethnicity, education level, and marital status.

^3^Adjusted for sex, age, race/ethnicity, education level, marital status, BMI, alcohol drinking status, smoking status, diabetes, hypertension, vigorous work activity, vigorous recreational activities, average daily caloric intake, and UIC.

### 3.5. Subgroup and Sensitivity Analysis

**FIGURE 3:**
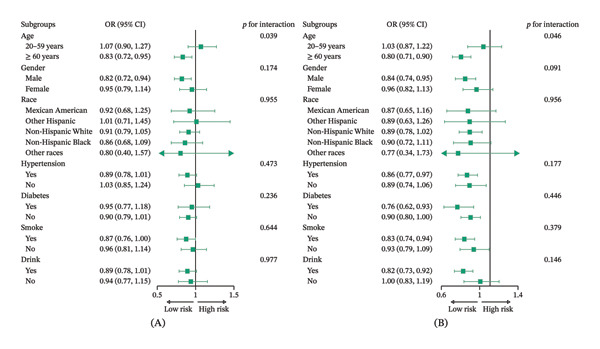
Subgroup analysis of the association between FT3, TT3, and CVD. (A) FT3; (B) TT3. Except for the stratifying variables, each subgroup analysis was adjusted for age, sex, ethnicity, smoking status, alcohol consumption status, hypertension, and diabetes.

Stratified analyses by age, sex, race, hypertension, diabetes, smoking, and alcohol consumption revealed significant interactions between FT3 and age group (*p*‐interaction = 0.039) and between TT3 and age group (*p*‐interaction = 0.046) (Figure 3).

To assess the robustness of our findings, we performed a sensitivity analysis using multiple imputation to address missing covariate data. Among participants aged ≥ 60 years, the results demonstrated that the direction, magnitude, and statistical significance of the key associations remained substantially unchanged. Detailed comparisons are provided in Supporting Tables S1–S5.

### 3.6. Mediation Analysis

The analysis revealed that PhenoAgeAccel significantly mediated 21.13% of the association between FT3 and CVD (*p* < 0.05) and 23.41% of the association between TT3 and CVD (*p* < 0.05). In the 20‐ to 59‐year group, no significant mediation was observed for either FT3 (*p* = 0.814) or TT3 (*p* = 0.936) (Table 5).

**TABLE 5:** Mediation analysis of PhenoAgeAccel in the associations of FT3 and TT3 with CVD.

Groups	OR (95% CI)				
	Total effect	Direct effect	Indirect effect	Mediation proportion (%)	*p* value
FT3					
Total	−0.00278 (−0.02681, 0.00543)	−0.00173 (−0.02448, 0.00788)	−0.00105 (−0.00461, −0.00039)	37.88	0.662
20–59 years	0.00179 (−0.00582, 0.01212)	0.00177 (−0.00592, 0.01214)	0.00002 (−0.00003, 0.00048)	1.34	0.814
≥ 60 years	−0.02969 (−0.05144, −0.01003)	−0.02342 (−0.04605, −0.00338)	−0.00628 (−0.01183, −0.00272)	21.13	0.004
TT3					
Total	−0.00437 (−0.02015, 0.00472)	−0.00262 (−0.01783, 0.00753)	−0.00175 (−0.00414, −0.00067)	40.10	0.446
20–59 years	−0.00064 (−0.00633,0.00638)	−0.00073 (−0.00656,0.00606)	0.00009 (−0.00018, 0.00060)	−14.56	0.936
≥ 60 years	−0.03013 (−0.05241, −0.00821)	−0.02308 (−0.04428, −0.00078)	−0.00705 (−0.01248, −0.00330)	23.41	0.004

*Note:* Models adjusted for age, sex, race/ethnicity, education level, marital status, BMI, alcohol drinking status, smoking status, diabetes, hypertension, vigorous work activity, vigorous recreational activities, average daily caloric intake, and UIC.

## 4. Discussion

Based on NHANES 2007–2010 data, this study explored the associations among FT3, TT3, CVD, and PhenoAgeAccel. The results showed that FT3 and TT3 were protective factors against CVD, and PhenoAgeAccel partially mediated this relationship, with a mediation proportion of approximately 20%. However, this mediating effect was significant only in individuals aged ≥ 60 years.

Most prior studies have focused on the direct association between TH levels and CVD. Higher FT4 levels increase the risks of all‐cause mortality, cardiovascular mortality, and CVD, whereas higher FT3 levels reduce these risks [22]. Ataoglu et al. reported that among long‐term hospitalized patients with nonthyroidal illness syndrome, low FT3 and high FT4 independently predicted long‐term mortality [23]. Thyroid aging is typically characterized by reduced thyroid volume and decreased circulating FT3 and FT4 levels. FT3 has emerged as a useful biomarker for identifying high‐risk older adults and evaluating frailty. Reduced FT3 levels predict increased mortality and play a critical role in the pathogenesis of frailty [24]. Zhang et al. found that low FT3 and TT3 levels are strong independent risk factors for frailty [25]. Age‐related changes in T3 and T4 follow similar trends, suggesting that both hormones may play a role in regulating lifespan. Higher T3 levels are linked to lower mortality, while higher T4 levels are associated with increased mortality. This discrepancy may be due to age‐dependent interactions between T3 and T4 [26].

In this study, the protective effects of FT3 and TT3 against CVD and the mediating role of PhenoAgeAccel were observed only in the elderly population, indicating an age‐dependent pattern. We propose that this phenomenon results from the combined effects of age‐related TH resistance and cumulative allostatic load (AL). This relationship can be explained from two aspects. First is age‐related TH resistance. Aging is accompanied by decreased sensitivity of peripheral tissues to TH, which is referred to as age‐related TH resistance [27, 28]. This form of age‐related TH resistance involves multiple mechanisms, including altered deiodinase activity, abnormal receptor signaling, and dysregulated gene expression, and typically manifests as tissue‐specific adaptive changes [29–31]. Its core mechanisms can be summarized as reduced TH transport into tissues, decreased nuclear receptor binding, impaired conversion of T4 to T3, and altered expression of TH‐responsive genes [28]. Age‐related resistance to THs is regarded as an adaptive physiological change that helps to extend lifespan [27, 32]. Taylor et al. further pointed out that normal thyroid status changes with advancing age, and that the aging process differentially affects the health effects of THs: Older individuals with lower thyroid function exhibit a survival advantage, whereas younger and middle‐aged individuals with low‐normal thyroid function face higher cardiometabolic risks [33]. Physiological changes in hypothalamic–pituitary–thyroid (HPT) axis function occur with aging, adjusting to reduced metabolic requirements and establishing a new balance between TH supply and metabolic demand [34].

Second, cumulative AL. AL is used to measure the cumulative biological burden experienced by the body while adapting to environmental and internal stressors. When adaptive responses are chronically overactivated, multisystem regulatory functions undergo cumulative “wear and tear,” leading to elevated AL [35]. In recent years, researchers have also focused on the association between AL and the endocrine axis. A cross‐sectional study based on the U.S. adult NHANES database showed that the allostatic load score (ALS) was significantly positively correlated with TSH levels and weakly positively correlated with FT3 levels. The association between ALS and TSH was more pronounced in young adults and weakened with advancing age [36]. Thyroid regulation is not a simple log‐linear feedback mechanism operating around a fixed setpoint, but rather a complex open system containing nonclassical mechanisms such as ultrashort autocrine loops and TSH–T3 shunting [37]. The HPT axis is a dynamic adaptive system with two allostatic modes: Type 1 is seen in energy‐deficient conditions such as illness and starvation, manifesting as low T3 syndrome—acute adaptation is beneficial, but chronic states easily lead to allostatic overload; Type 2 is seen in conditions of anticipated increased energy demand, such as pregnancy and obesity, manifesting as elevated TSH and T3 levels [38]. Elevated free TH concentration is a recognized cardiovascular risk factor. Psychosocial stress can raise the setpoint of the thyroid axis through Type 2 allostasis, resulting in elevated TSH levels without intrinsic thyroid dysfunction [39]. Although AL is generally associated with the incidence of CVD, its predictive value for mortality outcomes is limited. Furthermore, there is substantial heterogeneity across different CVD types and functional prognoses, and the predictive efficacy of AL is influenced by indicator composition and demographic characteristics [40]. Therefore, the age‐dependent vulnerability observed in this study is not due to a single mechanism but rather results from the synergistic effect of age‐related TH resistance and cumulative AL.

Thyroid function can influence biological aging by regulating the biochemical and hematological indicators incorporated into phenotypic age [16]. First, regarding inflammatory markers, high‐sensitivity C‐reactive protein (hsCRP) is a representative biomarker of systemic inflammation [41]. T3 inhibits the NLRP3 inflammasome via the nuclear receptor pathway, whereas T4 activates the NLRP3 inflammasome via the membrane receptor pathway; an imbalance between these two effects can lead to chronic inflammation and thyroid autoimmunity–related injury [42]. Priya et al. reported that in patients with subclinical hypothyroidism, hsCRP was significantly positively correlated with TSH and anti‐TPO antibodies, suggesting an association between thyroid autoimmunity and systemic inflammation [43]. In patients with clinical Hashimoto’s thyroiditis, RDW, mean platelet volume (MPV), and large platelet ratio (LPCR) are significantly positively correlated with high‐sensitivity C‐reactive protein (hs‐CRP) [44]. These lines of evidence indicate that changes in TH levels can affect CRP and RDW. Second, regarding hematological indicators, Haghbin et al. found that in hypothyroid patients, Hb and Hct were significantly correlated with T3, T4, and TSH, and MCH was significantly correlated with T3 and TSH, suggesting that THs primarily affect blood indicators by regulating erythropoiesis [45]. A case–control study also showed that thyroid dysfunction significantly affects multiple blood indicators, including red blood cell count, hemoglobin, and white blood cell count, with hypothyroidism‐related anemia being the most prominent [46]. Third, in addition to inflammatory and hematological markers, thyroid function is also associated with renal function. Meuwese et al. found in a cross‐sectional study that hypothyroidism was associated with lower eGFR [47]. Another study showed that compared with healthy controls, patients with subclinical hypothyroidism and clinical hypothyroidism had significantly higher serum creatinine levels, and the creatinine level in the clinical hypothyroidism group was higher than that in the subclinical hypothyroidism group [48]. Fourth, THs also play a critical role in metabolic regulation. In the context of insulin resistance and Type 2 diabetes, THs may improve glucose metabolism in cardiomyocytes by upregulating GLUT4, promoting mitochondrial function, and enhancing glucose oxidation [49]. T3 induces hepatocyte‐specific markers such as albumin [50]. Patients with hyperthyroidism have significantly elevated ALP levels [51]. Phenotypic age integrates chronological age with nine clinical biomarkers reflecting hepatic, renal, metabolic, inflammatory, and immune–hematological functions, thereby comprehensively capturing the extensive physiological imbalances related to the synthesis, metabolism, action, and pathogenesis of THs [52].

Our mediation analysis showed statistical associations among FT3, TT3, biological aging, and CVD, with PhenoAgeAccel playing a partial mediating role. This finding provides a new perspective for understanding the interaction mechanisms among THs, the aging process, and CVD. PhenoAgeAccel is widely regarded as an important predictor of age‐related morbidity and mortality [53]. Elevated PhenoAgeAccel is a significant risk factor for a range of adverse health outcomes, including CVD, cancer, depression, and all‐cause mortality [54]. As an emerging aging indicator, PhenoAgeAccel has recently received extensive attention because it can reflect an individual’s biological aging rate more accurately than chronological age [55]. Currently, emerging clinical trials focused on slowing or reversing the biological aging process may open new avenues for innovative management and prevention strategies for thyroid dysfunction. Although much work remains to be done, the overall prospect of improving the human aging process through effective means shows considerable potential.

Based on these results, we hypothesize that FT3 and TT3 may influence CVD risk by modulating the trajectory of PhenoAgeAccel, particularly in individuals aged ≥ 60 years. Future longitudinal studies are needed to test this hypothesis.

## 5. Limitations

This study has several limitations. First, as a cross‐sectional study, it cannot establish causal or temporal relationships. Second, the findings may not be generalizable beyond the U.S. population. Future studies should include longitudinal data and diverse ethnic groups. Third, self‐reported CVD may introduce recall bias. Fourth, despite adjustment for multiple confounders, residual confounding cannot be ruled out. Fifth, although the 2007–2010 data are the most recent cycle meeting all inclusion criteria, they are relatively old; however, the physiological associations studied are expected to remain stable over time.

## 6. Conclusion

Using mediation analysis on a nationally representative sample of U.S. adults, this study identified PhenoAgeAccel as a partial mediator in the associations of FT3 and TT3 with CVD. Age was a key modifier: higher FT3 and TT3 levels were associated with reduced PhenoAgeAccel among adults aged ≥ 60 years, thereby lowering CVD risk. These findings suggest that aging‐related mechanisms may represent a pathway through which thyroid function influences CVD risk in older adults. Assessing thyroid function and biological aging in older populations may offer new public health strategies for CVD prevention.

## Author Contributions

Wei Jie, Zhihua Shen, Minmin Wen, and Yanjun Hou: conceptualization; Minmin Wen and Yanjun Hou: writing–original draft; Minmin Wen, Yanjun Hou, Jiaxin Zuo, and Cheng Zhang: methodology; Minmin Wen, Kaijia Shi, and Shuya Zhang: formal analysis; Minmin Wen and Cheng Zhang: data curation; Wei Jie and Zhihua Shen: writing–review and editing; Wei Jie and Yanjun Hou: funding acquisition; Wei Jie and Zhihua Shen: supervision; Wei Jie: project administration. All authors reviewed the manuscript.

## Funding

The study was supported by National Natural Science Foundation of China (82260083, 82560052), the Academic Enhancement Support Program of Hainan Medical University (XSTS2025043) and the Hainan Province Clinical Medical Center.

## Disclosure

All data were generated in‐house, and no paper mill was used. The funders had no role in the design of the study, the collection, analysis, or interpretation of the data, or the preparation of the manuscript.

## Ethics Statement

Ethical approval for used human tissues was waived because this study used the data from publicly available databases.

## Consent

The authors have nothing to report.

## Conflicts of Interest

The authors declare no conflicts of interest.

## Supporting Information

Additional supporting information can be found online in the Supporting Information section.

## Supporting information


**Supporting Information** Supporting Table S1. Basic characteristics of the participants for the sensitivity analysis. Supporting Table S2. Associations of FT3 and TT3 with CVD among participants in the sensitivity analysis. Supporting Table S3. Associations of FT3 and TT3 with PhenoAgeAccel among participants in the sensitivity analysis. Supporting Table S4. Associations of PhenoAgeAccel with CVD in the sensitivity analysis. Supporting Table S5. Mediation analysis of PhenoAgeAccel in the associations of FT3 and TT3 with CVD in the sensitivity analysis.

## Data Availability

The datasets utilized and analyzed in this study were obtained from NHANES 2007–2010 (https://www.cdc.gov/nchs/nhanes/).
