# Supplementary material for: Phenotypic Age Acceleration as a Mediator in Thyroid Hormone–Related Cardiovascular Risk Among the Elderly
Source: Cardiol Res Pract. 2026 Jun 28;2026:1206757. doi: 10.1155/crp/1206757 (PMC13311311; doi:10.1155/crp/1206757)
Supplement: Supplementary file 1 — Supporting Information Supporting Table S1. Basic characteristics of the participants for the sensitivity analysis. Supporting Table S2. Associations of FT3 and TT3 with CVD among participants in the sensitivity analysis. Supporting Table S3. Associations of FT3 and TT3 with PhenoAgeAccel among participants in the sensitivity analysis. Supporting Table S4. Associations of PhenoAgeAccel with CVD in the sensitivity analysis. Supporting Table S5. Mediation analysis of PhenoAgeAccel in the associations of FT3 and TT3 with CVD in the sensitivity analysis. [file CRP-2026-1206757-s001.docx]

**Phenotypic Age Acceleration as a Mediator in Thyroid Hormone-Related Cardiovascular Risk among the Elderly**

Minmin Wen^1^, Yanjun Hou^1,3^, Kaijia Shi^1^, Jiaxin Zuo^1^, Cheng Zhang^1^, Shuya Zhang^1^, Zhihua Shen^2^, Wei Jie^1^

*^1^Key Laboratory of Tropical Translational Medicine of Ministry of Education & Hainan Provincial Key Laboratory for Tropical Cardiovascular Diseases Research, School of Public Health, Hainan Medical University, Haikou 571199, China*

*^2^Department of Pathophysiology, School of Basic Medicine Sciences, Guangdong Medical University, Zhanjiang 524023, China*

*^3^Department of Cardiovascular Surgery, The Second Affiliated Hospital, Hainan Medical University, Haikou 570100, China*

Minmin Wen and Yanjun Hou these authors contributed equally to this work.

Correspondence: Wei Jie (wei_jie@muhn.edu.cn); Zhihua Shen (shenzh@gdmu.edu.cn)

**Table S1. Basic characteristics of the participants for the sensitivity analysis**

|  | **Total** |  |  | **20-59 years** |  |  | **≥60 years** |  |  |
| --- | --- | --- | --- | --- | --- | --- | --- | --- | --- |
|  | **Non-CVD**  **(n=9695)** | **CVD**  **(n=1176)** | *P* | **Non-CVD (n=7018)** | **CVD**  **(n=312)** | *P* | **Non-CVD (n=2677)** | **CVD (n=864)** | *P* |
| Sex, n(%) |  |  | <0.001 |  |  | 0.061 |  |  | <0.001 |
| Male | 4,969 (51.25) | 725(61.65) |  | 3,614 (51.49) | 180(57.69) |  | 1355(50.62) | 545(63.08) |  |
| Female | 4,726(48.75) | 451(38.35) |  | 3,404 (48.51) | 132(42.31) |  | 1322 (49.38) | 319(36.92) |  |
| Age, years | 44.47±15.80 | 64.01±13.1 | <0.001 | 38.91± 11.28 | 48.69± 8.74 | <0.001 | 69.14± 6.82 | 72.42±6.70 | <0.001 |
| Race, n(%) |  |  | <0.001 |  |  | 0.006 |  |  | 0.741 |
| Mexican American | 1,832(18.90) | 142(12.07) |  | 1437 (20.48) | 42(13.46) |  | 395(14.75) | 100(11.57) |  |
| Other Hispanic | 1096(11.30) | 87(7.41) |  | 813 (11.58) | 23(7.37) |  | 283(10.57) | 64 (7.41) |  |
| Non-Hispanic White | 4344(44.80) | 656(55.78) |  | 2991(42.62) | 145(46.47) |  | 1353(50.54) | 511(59.14) |  |
| Non-Hispanic Black | 1926(19.87) | 249(21.17) |  | 1382(19.69) | 86(27.56) |  | 544 (20.32) | 163(18.87) |  |
| Other racess | 497(5.13) | 42(3.57) |  | 395(5.63) | 16(5.13) |  | 102(3.81) | 26(3.01) |  |
| Education, n (%) |  |  | <0.001 |  |  | <0.001 |  |  | <0.001 |
| ≤High School diploma | 5139(53.01) | 765(65.05) |  | 3582(51.04) | 207(66.35) |  | 1557(58.16) | 558(64.58) |  |
| >High School diploma | 4556(46.99) | 411(34.95) |  | 3436(48.96) | 105(33.65) |  | 1120(41.84) | 306(35.42) |  |
| Marriage,n (%) |  |  | <0.001 |  |  | <0.001 |  |  | 0.029 |
| Married | 5004(51.60) | 607(51.62) |  | 3,487 (49.69) | 147(47.12) |  | 1517(56.67) | 460(53.24) |  |
| Widowed | 663(6.84) | 245(20.82) |  | 90(1.28) | 14(4.49) |  | 573(21.40) | 231(26.74) |  |
| Divorced | 1043(10.75) | 156(13.27) |  | 709(10.10) | 50 (16.03) |  | 334 (12.48) | 106(12.27) |  |
| Separated | 338 (3.48) | 36(3.06) |  | 274(3.90) | 21(6.73) |  | 64(2.39) | 15(1.74) |  |
| Never married | 1870(19.28) | 93(7.91) |  | 1740(24.79) | 54(17.31) |  | 130(4.86) | 39(4.51) |  |
| Living with partner | 777(8.01) | 39(3.32) |  | 718(10.23) | 26 (8.33) |  | 59(2.20) | 13(1.50) |  |
| UIC, n (%) |  |  | <0.001 |  |  | 0.493 |  |  | 0.006 |
| <100 ug/L | 3,036 (31.33) | 328 (27.89) |  | 2,256 (32.16) | 91 (29.17) |  | 780 (29.13) | 237 (27.43) |  |
| 100-300 ug/L | 4,875 (50.31) | 547 (46.51) |  | 3,519 (50.17) | 149 (47.76) |  | 1,356 (50.64) | 398 (46.06) |  |
| ≥300 ug/L | 1,784 (18.41) | 301 (25.60) |  | 1,243 (17.72) | 72 (23.08) |  | 541 (20.21) | 229 (26.51) |  |
| Energy intake  (kcal/ day) | 2,145.45±859.61 | 1,866.26± 753.12 | <0.001 | 2,216.03± 884.07 | 1,988.13± 859.41 | <0.001 | 1,832.55± 655.80 | 1,799.21± 679.21 | 0.208 |
| BMI, kg/m2 | 28.37± 6.50 | 30.42± 7.23 | <0.001 | 28.29± 6.60 | 31.70±8.49 | <0.001 | 28.76± 6.01 | 29.59± 6.31 | 0.008 |
| TSH, mIU/L | 1.95± 2.18 | 2.06± 1.48 | 0.001 | 1.90± 2.13 | 1.95± 1.26 | 0.197 | 2.21± 2.25 | 1.99± 1.35 | 0.081 |
| FT3, pg/ml | 3.22± 0.48 | 3.07± 0.39 | <0.001 | 3.25±0.50 | 3.21±0.37 | 0.275 | 3.06±0.34 | 2.99± 0.38 | <0.001 |
| FT4, pmol/L | 10.02±1.87 | 10.28± 2.07 | 0.008 | 9.99± 1.90 | 10.13± 1.94 | 0.470 | 10.17± 1.72 | 10.35± 2.13 | 0.239 |
| TT3, ng/dL | 114.76± 23.21 | 107.30±23.30 | <0.001 | 116.10± 23.47 | 114.57± 23.67 | 0.475 | 108.79± 21.04 | 103.30± 22.11 | <0.001 |
| TT4,μg/dL | 7.77± 1.54 | 7.82± 1.54 | 0.290 | 7.75± 1.55 | 7.81± 1.46 | 0.534 | 7.82± 1.49 | 7.82± 1.58 | 0.703 |
| Hypertension, n (%) |  |  | <0.001 |  |  | <0.001 |  |  | <0.001 |
| Yes | 2834(29.24) | 859(73.04) |  | 1355(19.31) | 216(69.23) |  | 1479(55.26) | 643(74.42) |  |
| No | 6861(70.76) | 317(26.96) |  | 5663(80.69) | 96(30.77) |  | 1198(44.74) | 221(25.58) |  |
| Diabetes, n (%) |  |  | <0.001 |  |  | <0.001 |  |  | <0.001 |
| Yes | 934(9.63) | 374(31.80) |  | 400(5.70) | 81(25.96) |  | 534(19.96) | 293(33.91) |  |
| No | 8761(90.37) | 802(68.20) |  | 6618(94.30) | 231(74.04) |  | 2143(80.04) | 571(66.09) |  |
| Drink, n (%) |  |  | 0.108 |  |  | 0.393 |  |  | 0.861 |
| Yes | 7101(73.24) | 797(67.77) |  | 5362(76.40) | 236(75.64) |  | 1739(64.97) | 561(64.93) |  |
| No | 2594(26.76) | 379(32.23) |  | 1656(23.60) | 76(24.36) |  | 938(35.03) | 303(35.07) |  |
| Smoke, n (%) |  |  | <0.001 |  |  | <0.001 |  |  | 0.025 |
| Yes | 4381(45.20) | 738(62.82) |  | 3055(43.53) | 215(68.91) |  | 1326(49.53) | 523(60.53) |  |
| No | 5314(54.80) | 438(37.18) |  | 3963(56.47) | 97(31.09) |  | 1351(50.47) | 341(39.47) |  |
| Vigorous work activity, n (%) |  |  | <0.001 |  |  | 0.088 |  |  | 0.005 |
| Yes | 1,983 (20.45) | 120 (10.20) |  | 1,673 (23.84) | 54 (17.31) |  | 310 (11.58) | 66 (7.64) |  |
| No | 7,712 (79.55) | 1,056 (89.80) |  | 5,345 (76.16) | 258 (82.69) |  | 2,367 (88.42) | 798 (92.36) |  |
| Vigorous ecreational activities, n (%) |  |  | <0.001 |  |  | <0.001 |  |  | 0.022 |
| Yes | 2,078 (21.44) | 62 (5.27) |  | 1,877 (26.75) | 30 (9.62) |  | 201 (7.51) | 32 (3.70) |  |
| No | 7,617 (78.56) | 1,114(94.73) |  | 5,141 (73.25) | 282 (90.38) |  | 2,476 (92.49) | 832 (96.30) |  |
| PhenoAge (years) | 39.66± 17.76 | 64.30± 17.56 | <0.001 | 33.93± 13.40 | 48.60±14.79 | <0.001 | 65.04± 11.31 | 72.95± 12.12 | <0.001 |
| PhenoAgeAccel, n(%) |  |  | <0.001 |  |  | <0.001 |  |  | <0.001 |
| Yes | 4180(43.12) | 744(63.27) |  | 3,062 (43.63) | 203 (65.06) |  | 1,118 (41.77) | 541 (62.62) |  |
| No | 5515(56.88) | 432(36.73) |  | 3,956 (56.37) | 109(34.94) |  | 1,559 (58.23) | 323 (37.38) |  |

Continuous variables were presented as mean ± SD. Categorical variables were presented as n (%). Abbreviations: CVD, cardiovascular disease; BMI, Body Mass Index; TSH, Thyroid-Stimulating Hormone; FT3, free triiodothyronine; FT4, free triiodothyronine; TT3, total triiodothyronine; TT4, total thyroxine; PhenoAge, phenotypic age; PhenoAgeAccel, phenotypic age acceleration; UIC, urine iodine concentration

**Table S2. Associations of FT3 and TT3 with CVD among participants in the sensitivity analysis**

|  |  | **Model^1^** |  | **Model^2^** |  | **Model^3^** |  |
| --- | --- | --- | --- | --- | --- | --- | --- |
|  |  | **OR (95% CI)** | ***P*** | **OR (95% CI)** | ***P*** | **OR (95% CI)** | ***P*** |
| FT3 | Total | 0.316(0.253,0.394) | <0.001 | 0.775(0.608,0.987) | 0.040 | 0.797(0.595,1.068) | 0.109 |
|  | 20–59 years | 0.782(0.563,1.084) | 0.140 | 0.728(0.519,1.022) | 0.084 | 0.847(0.602,1.191) | 0.339 |
|  | ≥ 60 years | 0.511(0.372,0.702) | <0.001 | 0.508(0.370,0.699) | <0.001 | 0.544(0.392,0.755) | <0.001 |
| TT3 | Total | 0.984(0.979,0.989) | <0.001 | 0.994(0.989,0.999) | 0.026 | 0.996(0.990,1.001) | 0.103 |
|  | 20–59 years | 0.997(0.989,1.005) | 0.446 | 0.996(0.990,1.003) | 0.336 | 0.998(0.991,1.004) | 0.496 |
|  | ≥ 60 years | 0.987(0.982,0.992) | <0.001 | 0.988(0.984,0.993) | <0.001 | 0.990(0.985,0.994) | 0.002 |

Abbreviations: FT3, free triiodothyronine; TT3, Total Triiodothyronine; OR, Odds Ratio; CI, Confidence Interval; ^1^Crude model; ^2^Adjusted for sex, age, race/ethnicity, education level, marital status; ^3^Adjusted for sex, age, race/ethnicity, education level, marital status, BMI, alcohol drinking status, smoking status, diabetes, hypertension, vigorous work activity, vigorous recreational activities, average daily caloric intake, UIC.

**Table S3. Associations of FT3 and TT3 with PhenoAgeAccel among participants in the sensitivity analysis**

|  | Model^1^ |  | Model^2^ |  | Model^3^ |  |
| --- | --- | --- | --- | --- | --- | --- |
|  | β(95%CI) | *P* | β(95%CI) | *P* | β(95%CI) | *P* |
| FT3 | -1.239(-1.549,-0.929) | <0.001 | -1.320(-1.638,-1.002) | <0.001 | -1.235(-1.524,-0.946) | <0.001 |
| TT3 | -0.029(-0.036,-0.022) | <0.001 | -0.029(-0.036,-0.022) | <0.001 | -0.029(-0.036,-0.023) | <0.001 |

Abbreviations: FT3, free triiodothyronine; TT3, Total Triiodothyronine; CI, Confidence Interval. ^1^Crude model; ^2^Adjusted for sex, age, race/ethnicity, education level, marital status. ^3^Adjusted for sex, age, race/ethnicity, education level, marital status, BMI, alcohol drinking status, smoking status, diabetes, hypertension, vigorous work activity, vigorous recreational activities, average daily caloric intake, UIC.

| Outcome | Model^1^ |  | Model^2^ |  | Model^3^ |  |
| --- | --- | --- | --- | --- | --- | --- |
|  | OR (95% CI) | *P* | OR (95% CI) | *P* | OR (95% CI) | *P* |
| CVD | 1.050(1.045,1.063) | <0.001 | 1.040(1.035,1.053) | <0.001 | 1.020(1.016,1.035) | <0.001 |

**Table S4. Associations of PhenoAgeAccel with CVD in the sensitivity analysis**

Abbreviations: CVD, cardiovascular disease; OR, Odds Ratio; CI, Confidence Interval; ^1^Crude model; ^2^Adjusted for sex, age, race/ethnicity, education level, marital status; ^3^Adjusted for sex, age, race/ethnicity, education level, marital status, BMI, alcohol drinking status, smoking status, diabetes, hypertension, vigorous work activity, vigorous recreational activities, average daily caloric intake, UIC.

**Table S5. Mediation analysis of PhenoAgeAccel in the associations of FT3 and TT3 with CVD in the sensitivity analysis**

|  |  | OR (95% CI) |  |  |  |
| --- | --- | --- | --- | --- | --- |
| Groups | Total Effect | Direct effect | Indirect effect | Mediation  Proportion（%） | p value |
| FT3 |  |  |  |  |  |
| Total | -0.00387(-0.01610, 0.00380) | -0.00272(-0.01510, 0.00525) | -0.00116( -0.00248, -0.00060) | 29.90 | 0.426 |
| 20-59 years | -0.00222(-0.00830, 0.00251) | -0.00213(-0.00818, 0.00261) | -0.00009( -0.00027, -0.00000) | 4.198 | 0.570 |
| ≥ 60 years | -0.02866(-0.04055,-0.01392) | -0.02297(-0.03595,-0.00840) | -0.00568(-0.00889, -0.00349) | 19.84 | < 0.001 |
| TT3 |  |  |  |  |  |
| Total | -0.00326(-0.01101, 0.00409) | -0.00189(-0.01007, 0.00563) | -0.00136(-0.00225, -0.00074) | 41.88 | 0.408 |
| 20-59 years | -0.00179( -0.00615, 0.00261) | -0.00164(-0.00602, 0.00277) | -0.00015(-0.00030, 0.00001) | 8.14 | 0.540 |
| ≥ 60 years | -0.02920(-0.04284, -0.01540) | -0.02467(-0.03859,-0.01039) | -0.00453(-0.00713, -0.00258) | 15.50 | < 0.001 |

Models adjusted for age, sex, race/ethnicity, education level, marital status, BMI, alcohol drinking status, smoking status, diabetes, vigorous work activity, vigorous recreational activities, average daily caloric intake, UIC and hypertension
